# Enquete sur les aspects toxicologiques de la phytotherapie utilisee par un herboriste à Fes, Maroc

**DOI:** 10.11604/pamj.2013.14.125.1746

**Published:** 2013-03-30

**Authors:** Ali Amine Zeggwagh, Younes Lahlou, Yassir Bousliman

**Affiliations:** 1 Service de Réanimation Médicale et de Toxicologie Clinique-Faculté de Médecine et de Pharmacie de Rabat - Maroc; 2 Laboratoire de Pharmacologie et Toxicologie de Rabat, Faculté de Médecine et de Pharmacie de Rabat-Maroc

**Keywords:** Ethnobotanique, Maroc, Phytothérapie, Plantes médicinales, Toxicité, Ethnobotany, Morocco, Phytotherapy, medicinal plants, toxicity

## Abstract

**Introduction:**

Dans le but d'étudier l'aspect toxicologique des plantes médicinales utilisées en médecine traditionnelle, une étude ethnobotanique a été réalisée à la ville de Fès au centre du Maroc.

**Méthodes:**

Ont été inclus dans l'étude tous les patients ayant bénéficié d'une prescription par l'herboriste de plantes à visée thérapeutique. La discussion de nos résultats s'est faite sur la base d'une revue de la littérature avec identification des principales plantes toxiques utilisées en phytothérapie au Maroc. L'approche bibliographique a permis de compléter les informations.

**Résultats:**

L'âge moyen des patients traités par des plantes (38 femmes, 32 hommes) était de 35 ± 18 ans. L'enquête ethnobotanique à révélé que la majorité des plantes médicinales étaient utilisées contre les affections urinaires (21%), suivi des maladies de l'appareil digestif (19.6%) et des maladies rhumatologiques (18.2%). Le nombre de plantes prescrits par l'herboriste a été de 53 dont 5 sont potentiellement toxiques. L'identification taxonomique des plantes prescrites a recensé 30 familles dont les plus représentées sont les *Lamiaceae* (23.33%), les *Apiaceae* (13,33%) et les *Asteraceae* (10%). La prescription des plantes considérées comme toxiques a concerné 7,1% des consultants traités par les plantes médicinales. Aucune complication inhérente aux plantes prescrites n'a été déplorée.

**Conclusion:**

Malgré les résultats encourageants de notre enquête sur le compte de la phytothérapie, la pratique de la phytothérapie est laissée à la vulgarisation et à l'oubli scientifique, législatif et universitaire.

## Introduction

Selon l'OMS, dans certains pays en voie de développement d'Asie, d'Afrique et d'Amérique latine, 80% de la population dépend de la médecine traditionnelle surtout en milieu rural du fait de la proximité et de l'accessibilité de ce type de soins au coût abordable et surtout en raison du manque d'accès à la médecine moderne de ces populations [1–4]. De ce fait, la médecine traditionnelle peut être considérée comme partie intégrante des soins de santé primaire, pour améliorer l'accès aux soins. Ainsi, il faut évaluer l'efficacité clinique, assurer la sécurité des plantes médicinales, renforcer les connaissances et les performances des herboristes tradithérapeutes et garantir un suivi suffisant des patients. Un regain d'intérêt envers la phytothérapie durant ces dernières années a permis d'approfondir l'analyse de son efficacité thérapeutique et surtout de son aspect toxicologique [5]. Ce dernier aspect reste en retrait par rapport à l'avancement de la phytothérapie. En effet, plusieurs études réalisées sur les traitements traditionnels à base de plantes ont fait état de problème de toxicité ou d'interaction pouvant causer des échecs thérapeutiques ou des accidents [6]. D'autre part, les principes toxiques des plantes sont peu connus, essentiellement du fait de leur complexité naturelle. Ces plantes médicinales doivent alors, comme les « médicaments conventionnels », obéir à des règles strictes de culture, de contrôle et de dispensation. Actuellement, 205 drogues végétales entrent dans la composition de médicaments dits de phytothérapie et bénéficient d'un dossier allégé d'autorisation de mise sur le marché [5]. En 2007, 62 pays avaient des instituts de médecine traditionnelle, contre 12 en 1970 [7]. L'Organisation Mondiale de la Santé a établi une liste de monographies de plantes médicinales qui est divisée en trois catégories: plantes dont l'utilisation est supportée par des données cliniques, celles dont l'utilisation est supportée par des pharmacopées et des systèmes traditionnels de médecine et celles dont l'utilisation est décrite dans le milieu populaire mais non supportée par des données cliniques et expérimentales [8].

L'Afrique est réputée pour la richesse de sa flore d'où la nécessité de prendre les initiatives pour une meilleure valorisation de ces ressources naturelles. Le Maroc, possédant une richesse importante en plantes avec environ 42000 espèces dont près de 600 utilisées en médecine traditionnelle [9], connaît une incidence non négligeable des intoxications issues des plantes (5.1%) [10]. L'objectif de ce travail était de réaliser une enquête sur le terrain auprès d'un herboriste afin de préciser la nature et la proportion de prescription des plantes supposées toxiques et de recueillir l'ensemble des informations sur les effets chez les consommateurs de ces plantes médicinales.

## Méthodes

Il s'agissait d'une étude prospective de cohorte longitudinale étalée sur une période d'un mois. L'enquête a été réalisé à Fès (Centre du Maroc) chez un herboriste ayant obtenu son certificat d'herboriste en Egypte et effectué des stages en France pour la maîtrise de matériaux utilisés dans l'extraction des huiles essentielles et en Hollande pour l'étude des dosages en phytothérapie. Ont été inclus tous les patients ayant bénéficié au cours de l'étude d'une prescription par l'herboriste de plantes à visée thérapeutique. Ont été exclus les patients à qui l'herboriste a prescrit des traitements à base de minéraux et de produits animaux seuls ou en association aux plantes et toute personne ayant consulté pour des motifs non thérapeutiques. Après entretien avec les individus, l'herboriste prescrivait une thérapeutique sur la base des symptômes rapportés et observés. Plusieurs variables ont été recueillis sur une fiche d'exploitation individuelle: identité, âge, sexe, motif de consultation, diagnostic évoqué, traitement proposé avec son mode d'administration, sa dose, sa durée et son coût. Concernant les données évolutives, les patients étaient contactés à la fin du traitement à leur domicile ou par téléphone afin de préciser leur amélioration ou leur guérison, la survenue de complications de même que leur survie ou leur décès.

Certaines plantes non toxiques peuvent avoir un effet nocif sur divers organes humains ou animaux, du fait de leur emploi à des doses excessives ou de leur absorption pendant une longue durée. La discussion de nos résultats s'est faite sur la base d'une revue de la littérature avec identification essentiellement des principales plantes toxiques utilisées en phytothérapie au Maroc [11–13].

## Résultats

Durant la période étudiée, 90 patients ont consulté l'herboriste dont 7.8% ont été traités par des minéraux (7 cas), 3.3% par des produits animaux (3 cas), 11.1% par un mélange de plantes, de minéraux et de produits animaux (10 cas) et 77.8% par des plantes seules (70 cas).

L'âge moyen des patients (38 femmes, 32 hommes) était de 35 ±; 18 ans (extrêmes: 7 - 93 ans). La plupart des patients étaient originaires des quartiers populaires de Fès, et, plus rarement, des régions et villes avoisinantes. Le motif de consultation a été des symptômes isolés ou bien entrant dans le cadre d'un syndrome bien défini ou encore une maladie diagnostiquée au préalable par un médecin. L'enquête ethnobotanique à révélé que la majorité des plantes médicinales étaient utilisées contre les affections urinaires (21%), suivi des maladies de l'appareil digestif (19.6%), des maladies rhumatologiques (18.2%) et des maladies dermatologique (16.8%). Le reste des maladies (pulmonaire, gynécologique, auditif, système nerveux et autres) représentaient 24.4% (Tableau 1).

**Table 1:** Motifs de consultation (n = 70)

Motif de consultation	Pourcentage %	Symptômes
Urinaire	21	Brûlures mictionnelles, dysurie, polyurie, énurésie, incontinence urinaire, adénome de prostate.
Digestif	19,6	Epigastralgie, ballonnement abdominal, vers intestinaux, intoxication alimentaire, hémorroïdes, kyste hydatique
Rhumatologique	18,2	Arthralgies, lombalgies, dorsalgies
Dermatologique	16,8	Chute ou sécheresse des cheveux, acné, sécheresse de la peau, plaies, hyperpigmentation, eczéma, psoriasis, goutte matinale
Respiratoire	9,8	Bronchite aigue et chronique
Gynécologique	5,6	Aménorrhée, dysménorrhée, mastodynie, stérilité
Oto-rhino-laryngologique	2,8	Pharyngite, rhinorrhée, enrouement
Autres	6,2	Amaigrissement, céphalées, désir d'arrêter de fumer.

Quatorze individus (20%) ont été traités par des huiles essentielles, 7 par des plantes uniques (10%) et 49 par l'association de deux plantes ou plus (70%). Les préparations de plantes ont été administrées: 1) par voie interne (ingestion orale, lavements) chez 46 individus (65.71%): poudre, mélange avec le miel, hydrolat, infusion, décoction, huile, extraits, sirop, gouttes; 2) par voie externe (application locale) chez 22 individus (31.42%): pommade, cataplasme, masque, compresse, lotion, collyre, fumigation, gouttes auriculaires; et 3) l'association de la voie orale et externe a été prescrite chez 2 individus (2.85%).

Le coût moyen du traitement a été de 9.6 ± 8.2 euros avec des extrêmes de 1 euro (traitement complet d'une bronchite) et 60 euros (traitement nécessitant des préparations particulières).

Le Tableau 2 représente la répartition des plantes médicinales recensées en fonction de leurs indications thérapeutiques. Le nombre de plantes prescrites par l'herboriste a été de 53 dont:

**Table 2:** Répartition des plantes médicinales recensées en fonction de leurs indications thérapeutiques

Indications	Plantes
Digestive	*Artemisiae absinthium, Artemisia vulgaris, Ceratonia siliqua, Cinnamomum, Zeylanicum, Citrullus colocynthis, Citrus limonia, Cocos nucifera, Coriandrum, Sativum, Crocus sativus, Fragaria vesca, Mentha piperita, Myristica fragrans, Myrtus communis, Nigella sativa, Ocymum basilicum, Ormenis mixta, Papaver, Somniferum, Peganum harmala, Phoenix dactylifera, Pimenta officinalis, Pimpinella anisum, Prunus amygdalus, Punica granatum, Quercus suber, Raphanus sativus, Ricinus communis, Rosa centifolia, Rosmarinus officinalis, Ruta graveolens, Salvadora persica, Sesamum indicum, Thymus serpyllum, Thymus vulgaris, Trigonnella foenum-graecum, Vitis vinifera, Zea mays, Zingiber Officinale*.
Respiratoire	*Atropa belladona, Brassica rapa,Rosmarinus officinalis, Viola odorata, Cinnamomum zeylanicum, Eugenia cariophyllata,Mentha piperita, Marrubium vulgare, Myristica fragrans, Myrtus communis, Ocymum basilicum, Ormenis mixta, Papaver rhoeas, Papaver somniferum, Petroselinum sativum,Phoenix dactylifera, Pimpinella anisum, Prunus amygdalus, Thymus serpyllum, Thymus vulgaris, Zingiber officinale*.
Cardio-vasculaire	*Allium sativum, Argania spinosa,Artemisia vulgaris, Atropa belladona, Brassica rapa, Cinnamomum zeylanicum, Corylus avellana, Nigella sativa, Olea europaea, Papaver rhoeas, Pimenta officinalis, Punica Granatum, Rosmarinus officinalis, Sesamum indicum, Thymus vulgaris, Trigonnella foenum-graecum, Viola odorata, Vitis vinifera, Prunus amygdalus*.
Neurologique	*Atropa belladona, Citrullus colocynthis, Lavandula officinalis, Malus pumila, Mentha piperita, Myrtus communis, Ormenis mixta, Papaver rhoeas, Papaver somniferum, Peganum harmala, Petroselinum sativum, Pimenta officinalis, Prunus amygdalus, Rosa centifolia, Ruta graveolens, Sesamum indicum, Thymus vulgaris*.
Dermatologique	*Argania spinosa, Citrus limonia, Cocos nucifera, Corylus avellana, Lawsonia inermis, Peganum harmala, Pimenta officinalis, Prunus amygdalus, Quercus suber, Raphanus sativus, Rosa centifolia, Ruta graveolens, Sesamum Indicum*.
Gynécologique	*Citrullus colocynthis, Ocymum basilicum, Ormenis mixta,Papaver somniferum, Peganum harmala, Petroselinum sativum, Pimpinella anisum, Ricinus communis, Rosa centifolia, Ruta graveolens, Zingiber officinale*.
Urinaire	*Atropa belladonna, Citrullus colocynthis, Myrtus communis, Ocymum basilicum, Olea europaea, Petroselinum sativum, Phoenix dactylifera, Punica granatum, Raphanus sativus, Zea mays*.
Rhumatologique	*Cinnamomum camphora, Citrus limonia, Myristica fragrans, Papaver somniferum, Rosmarinus officinalis, Ruta graveolens, Thymus vulgaris, Vitis vinifera, Zea mays, Zingiber officinale*.

5 plantes potentiellement toxiques (9,3%): *Atropa belladona* La Belladone (Nom Arabe: Belaydour), *Citrullus colocynthis* le Coloquinte (Nom Arabe: Hdej), *Papaver somniferum* le Pavot (Nom Arabe: Kharchacha), Peganum harmala le Harmel, *Ruta graveolens* la Rue (Nom Arabe: Figel) 48 plantes non toxiques pouvant devenir toxiques sous certaines conditions d'utilisation: *Allium sativum* (Ail), *Argania spinosa* (Arganier), *Artemisiae absinthium* (Absinthe), *Artemisia vulgaris* (armoise), *Brassica rapa* (Navet), *Ceratonia siliqua* (Caroube), *Cinnamomum camphora* (huile camphrée), *Cinnamomum zeylanicum* (Cannelle), Citrus *limonia* (Citron), *Cocos nucifera* (Noix de coco), *Coriandrum sativum* (Coriandre), *Corylus avellana* (Noisette), *Crocus sativus* (Safran), *Daucus carota* (Carotte), *Eugenia cariophyllata* (Clou de girofle), *Fragaria vesca* (Fraise), *Lavandula officinalis* (lavande), *Lawsonia inermis* (Henné), *Malus pumila* (Pomme), *Marrubium vulgare* (Marrube), *Mentha piperita* (menthe), *Myristica fragrans* (Muscadier), *Myrtus communis* (Myrte), *Nigella sativa* (Nigelle), *Ocymum basilicum* (Basilic), *Olea europaea* (Olivier), *Ormenis mixta* (Camomille), *Papaver rhoeas* (Coquelicot), *Petroselinum sativum* (Persil), *Phoenix dactylifera* (Dattes), *Pimenta officinalis* (Piment de Jamaïque), *Pimpinella anisum* (Anis), *Prunus amygdalus* (Amande douce), *Punica granatum* (Grenadier), *Quercus suber* (Chêne), *Raphanus sativus* (Radis), *Ricinus communis* (Ricin), *Rosa centifolia* (Rose), *Rosmarinus officinalis* (Romarin), *Salvadora persica* (Souak), *Sesamum indicum* (Sésame), *Thymus serpyllum* (Serpolet), *Thymus vulgaris* (Thym), *Trigonnella foenum-graecum* (Fénugrec). *Viola odorata* (Violette), *Vitis vinifera* (Raisin sec), *Zea mays* (maïs), *Zingiber officinale* (Gingembre).

L'identification taxonomique des plantes prescrites a recensée 30 familles dont les plus représentées sont les Lamiaceae (23.33%), les Apiaceae (13.33%) et les Asteraceae (10%). Les autres familles restantes ne comportaient qu'une à deux espèces (53.33%). La prescription de plantes considérées comme toxiques a donc concerné 5 patients soit 5.5% de l'ensemble des sujets ayant consulté durant le mois et 7.1% des consultants traités par les plantes. Les plantes non toxiques ont été utilisées seules dans 90% des cas et associées aux plantes toxiques dans 10% des cas.

L'évolution a été déclarée favorable chez 59 patients (84.28%) sous forme d'amélioration de la symptomatologie ou de guérison. Chez 11 patients (15.72%), aucune amélioration n'a été notée. Aucune complication inhérente aux plantes prescrites n'a été déplorée.

## Discussion

### Usage de la phytothérapie

La présente étude, relative à l'étude de la phytothérapie utilisée dans le cadre de la médecine traditionnelle, a inclus le suivi des prescriptions d'un tradipraticien herboriste diplômé et qualifié. La prescription de plantes toxiques dans certaines préparations a concerné 7.1% des consultants et a été justifiée par l'herboriste par le respect des règles spécifiques des préparations par ajouts d'antagonistes aux toxicités et par adaptation des doses des plantes réputées toxiques comme le stipule le dicton de Paracelse «rien n'est poison, tout est poison: seule la dose fait le poison ». Ceci est réconforté par l'absence de toute complication ou intoxication suite aux prescriptions traditionnelles dans notre étude. Différentes plantes peuvent être réputées à la fois comme toxiques et plantes médicinales. La mise en évidence de la toxicité de la phytothérapie n'est pas toujours aisée. Ainsi, d'après De Smet, pour avoir 95 chances sur cent d'observer 3 fois une réaction secondaire à la phytothérapie qui se traduit chez un patient sur 1000, un praticien doit avoir au moins 6500 patients [14].

### Fréquences et raisons de l'utilisation des plantes médicinales

La phytothérapie est fréquemment pratiquée par la population marocaine. En effet, des études ont montré que, selon les régions, 55 à 90% des patients utilisent des plantes pour traiter des maladies chroniques notamment le diabète, l'hypertension et les maladies urinaires [15]. Dans la région de Fès où notre enquête a été menée, 68% à 76% des personnes affirmaient utiliser régulièrement les plantes médicinales pour le traitement des maladies particulièrement le diabète [16]. Selon des études ethnobotaniques marocaines, 365 espèces de plantes sont utilisées comme alimentaires, médicinales, aromatiques, condimentaires et toxiques et 500 préparations utilisées pour soigner différentes pathologies sont à base de plantes [5].

Selon une étude, 70 à 80% des marocains font appel aux plantes médicinales pour se faire soigner, 60% d'entre eux sont de sexe féminin et plus de 50% sont analphabètes et âgées de plus de 50 ans [15].

Une étude floristique et ethnobotanique des plantes médicinales dans une autre ville du Maroc a recensée comme dans notre étude les mêmes familles de plantes les plus représentées en médecine traditionnelle à savoir les *Lamiaceae*, les *Asteraceae* et les *Apiaceae* [17]. L'ensemble des plantes dénombrées sont connues universellement dans leurs propriétés phytochimique et pharmacologique et figure dans la pharmacopée marocaine [7].

Concernant les motifs d'accès des populations à ce type de thérapie, une étude portant sur l'usage des plantes médicinales dans le traitement du diabète au Maroc intéressant 356 patients a relaté que 77% des patients déclaraient comme principales raisons pour l'usage des plantes médicinales, l'expérience, jugée positive, d'un autre malade, 44% pour réduire la part des traitements médicamenteux, 17% en tant que complément thérapeutique et 13% ont recours aux plantes en raison de leur faible coût. L'ensemble des 356 patients enquêtés ignoraient toutes informations sur la toxicité des plantes utilisées [18].

### Prescripteurs

En matière de la qualité des tradipraticiens que les patients consultent on peut distinguer:

- les herboristes, diplômés ou non, qui sont capables de reconnaître, cueillir et vendre des plantes médicinales. Ils connaissent bien les plantes et peuvent contribuer au diagnostic des maladies et prescrire les traitements appropriés.
- les droguistes qui sont des commerçants vendeurs de produits alimentaires, condimentaires, cosmétiques et de plantes médicinales.
- les guérisseurs qui sont généralement dépourvues de diplôme médical et prétendent guérir en utilisant des pratiques magiques ou religieuses associant des fois la phytothérapie. Dans certains pays, les guérisseurs risquent des poursuites judiciaires pour exercice illégal de la médecine [19].

D'après une étude réalisée sur 15 villes marocaines auprès de 2000 personnes, l'approvisionnement en plantes médicinales se fait en premier lieu des droguistes (98.4%), suivi des herboristes (17.7%), des pharmaciens (8.1%) et en dernier lieu des guérisseurs (5.8%) [9].

### Plantes médicinales et toxicité

Au Maroc, les intoxications par les plantes représentent 5.1% de toutes les intoxications en dehors des piqures et envenimation scorpioniques, dont les trois premières plantes sources d'intoxication végétale sont le Chardon à glu, *Atractylis gummifera* (10.1%), le Cannabis, *Cannabis sativa* (4.6%) et le Harmel, *Peganum harmala* (3.6%) [10]. Une enquête ethnobotanique des plantes médicinales réalisée dans la même région que notre étude, Fès, a révélée que seulement 12% des patients annoncent qu'ils ont une connaissance des plantes toxiques [20].

Des études sur les effets indésirables de la phytothérapie montrent que la plupart des effets nocifs des plantes médicinales sont rapportés non pas à la plante elle-même, mais à une erreur d'identification, à une contamination involontaire (par une autre plante, par des métaux lourds, par des micro-organismes pathogènes ou par des résidus agrochimiques), à un non respect de la dose adéquate ou à une interaction avec les médicaments. Atitre d'exemple, le ginseng a peu d'effets négatifs graves quand il est pris seul, toutefois, s'il est combiné avec la warfarine, son activité antiplaquettaire risque d'entrainer une anticoagulation excessive [21]. Pour l'exemple de la contamination des plantes, on peut citer le cas de l'ail, souvent utilisé pour réduire le taux de cholestérol, qui peut ne pas produire de tels effets s'il est transformé de certaines manières [2, 22]. L'erreur d'identification des plantes peut être illustré par l'exemple de Belgique ou plus de 50 personnes ont été atteintes d'insuffisance rénale en 1996 après avoir ingéré une préparation à base de plantes contenant *Aristolochia fangchi* (guang fang ji), une plante toxique, au lieu de *Stephania tetrandra* (fang chi hang) suite à la confusion entre ces deux espèces portant des noms vernaculaires chinois très proche [16, 23]. L'effet nocif des remèdes à base de plante peut dépendre aussi de facteurs liés aux consommateurs, tels que l'âge, la génétique et les maladies concomitantes [24].

### Approche réglementaire

La réglementation de la profession d'herboristerie au Maroc se base sur des textes de loi datant de 1923, 1926, et 1960 qui interdisent aux herboristes de mettre en vente toute plante vénéneuse ou toxique [25]. Une enquête menée en 2005 auprès des pays membres de l'OMS a conclu qu'entre 80 et 90 pays n'ont pas de politique nationale dans le domaine de la médecine à base de plantes médicinales [26]. Cette constatation concerne souvent les pays où les pratiques traditionnelles sont les plus utilisées, où l'évolution de la phytothérapie a été influencée par le contexte culturel et historique et où il y a absence d'évaluation de l'innocuité et de l'efficacité de ces plantes, ce qui a freiné la mise en place d'une réglementation et d'une législation [26].

### Limite de l'étude

La limite de notre étude vient du fait qu'elle ne prend en compte que les prescriptions d'un seule herboriste. Cependant, le nombre élevé des plantes prescrites (53 espèces appartenant à 30 familles botaniques) a permis d'élaborer un recueil de données intéressant des plantes médicinales. La représentativité des espèces colligées a été réconforté par une étude ethnobotanique marocaine dans la ville de Kenitra sur 200 personnes qui a permis d'inventorier presque le même nombre de plantes médicinales (55 espèces) appartenant à peu près aux mêmes nombre de familles (32 familles botaniques) en outre, l'identification botanique à recensée les mêmes familles de plantes les plus représentées que dans notre série à savoir les *Lamiaceae*, les *Asteraceae* et les *Apiaceae* [17]. Au même titre une autre étude ethnobotanique dans la province d'Essaouira au Maroc a montré que la population de la région utilisait 42 espèces de plantes appartenant à 29 familles dont les plus représentées sont également les *Lamiaceae* suivi des *Asteraceae* [27].

## Conclusion

La phytothérapie utilise des produits biologiques ayant des propriétés pharmacodynamiques bien précises et pouvant induire des incidents toxiques très importants. La cause de ces intoxications d'origine végétale est essentiellement accidentelle, volontaire et criminelle. Malgré les résultats encourageants de notre enquête sur le compte de la phytothérapie, la pratique de cette dernière au Maroc est laissée à la vulgarisation et à l'oubli scientifique, législatif et universitaire. Les plantes médicinales, doivent, comme les médicaments, obéir à des règles standard strictes auxquelles seul le spécialiste en phytothérapie peut répondre. Ceci implique forcément la réglementation de la profession dans notre pays.
